# Supplementary material for: Mapping the risk of Rift Valley fever in Uganda using national seroprevalence data from cattle, sheep and goats
Source: PLoS Negl Trop Dis. 2023 May 26;17(5):e0010482. doi: 10.1371/journal.pntd.0010482 (PMC10249900; doi:10.1371/journal.pntd.0010482)
Supplement: S1 Table — (DOCX) [file pntd.0010482.s001.docx]

| **Description** | **Units** | **Source** | **Resolution** | **Production** |
| --- | --- | --- | --- | --- |
| Actual evapotranspiration | Mm | https://figshare.com/articles/Global_High-Resolution_Soil-Water_Balance/7707605/3 | 30 m | This model uses spatially distributed values (average over the 1950-2000 period) of monthly precipitation (Prec) and monthly Potential Evapotranspiration (PET) and returns monthly spatially-distributed values defining Actual Evapotranspiration (AET), Runoff (R), and Soil Water Content (SWC) [1] |
| Annual relative humidity | percentage (%) | CRU:  https://crudata.uea.ac.uk/cru/data/hrg/tmc/ | ~55 km | It was interpolated from a data set of station means for the period cantered on 1961 to 1990. The number of stations for each variable varies markedly [2] |
| Digital elevation model | angles (degrees) | USGS https://www.usgs.gov/publications/new-landsat-collection-2-digital-elevation-model | 30 m | In the data processing phase, the USGS uses algorithms and advanced statistical techniques to interpolate the elevation data and create a continuous surface model. This model is then checked for accuracy and quality using various methods, including visual inspection and comparison with other datasets.  The product is available worldwide with few exceptions [3] |
| Mean annual temperature | ◦c*10 | University of York, AfriClim:  https://webfiles.york.ac.uk/KITE/AfriClim/GeoTIFF_30s/baseline_worldclim/ | 25 km | Downscaled monthly temperature, wind speed, humidity and rainfall variables from 1950 to present and calculated using standard formulas. |
| Mean temp warmest quarter | ◦c*10 |  | 25 km |  |
| Mean annual rainfall | Mm |  | 25 km |  |
| Rainfall wettest month | Mm |  | 25 km |  |
| Precipitation of driest Month | Mm |  | 25 km |  |
| Rainfall seasonality | Mm |  | 25 km |  |
| Rainfall wettest quarter | Mm |  | 25 km |  |
| Rainfall driest quarter | Mm |  | 25 km |  |
| Mean diurnal range in temp | ◦c*10 |  | 25 km |  |
| Temperature seasonality | ◦c*10 |  | 25 km |  |
| Max temp warmest month | ◦c*10 |  | 25 km |  |
| Min temp coolest month | ◦c*10 |  | 25 km |  |
| Annual temperature range | ◦c*10 |  | 25 km |  |
| Number of dry months | months |  | 25 km |  |
| Length of longest dry season | months |  | 25 km |  |
| Annual moisture index | index |  | 25 km |  |
| Moisture index moist quarter | index |  | 1 km |  |
| Potential evapotranspiration | Mm |  | 25 km | The data are produced using the Joint Research Centre's own meteorological and land surface models, which are based on observations from a range of sources, including satellites, weather stations, and reanalysis datasets. The models are calibrated using observations of PET and other meteorological variables and are designed to represent the spatial and temporal variability of PET across Africa. The resulting data are presented in a consistent and standardized format, making them easily accessible and widely applicable. |
| Isothermality | ◦c*10 | University of York, AfriClim:  https://webfiles.york.ac.uk/KITE/AfriClim/GeoTIFF_30s/baseline_worldclim/ | 25 km | In the Africlim dataset, isothermality is calculated as the ratio of the mean diurnal temperature range (the difference between the daily maximum and minimum temperatures) to the mean annual temperature. It is presented as a percentage, with higher values indicating a larger temperature range and lower values indicating a smaller temperature range. The isothermality data in the Africlim dataset can be used to understand the temperature variability within a region and to identify areas with similar climate conditions. |
| Soil clay content (0-2 micrometre) at depth 0.00 m (Clay) (ISRIC) | mass fraction (%) | ISRIC:  https://files.isric.org/soilgrids/former/2017-03-10/data/ | 250 m | The samples consist of all physically archived samples at ISRIC in 2004 for which soil attribute data was available  The ISRI soil data are collected from a range of sources, including field observations, laboratory analyses, and remote sensing data. The data are then processed and standardized. |
| Soil silt content (0-2 micrometre) at depth 0.05 m | mass fraction (%) |  | 250 m |  |
| Sand content (0-2 micrometer) at depth 0.05 m | mass fraction (%) |  | 250 m |  |
| Soil pH x 10 in H2O at depth 0.05 m ( | mass fraction (% |  | 250 m |  |
| Soil texture fraction at depth 0.00 m | factor |  | 250 m |  |
| Albic luvisols | % |  | 250 m |  |
| Calcic vertisols | % |  | 250 m |  |
| Calcic luvisols | % |  | 250 m |  |
| Endogleyic panosols | % |  | 250 m |  |
| Gleyic luvisols | % |  | 250 m |  |
| Gypsic solonchaks | % |  | 250 m |  |
| Haplic calcisols sodic | % |  | 250 m |  |
| Haplic calcisols | % |  | 250 m |  |
| Haplic luvisols ferric | % |  | 250 m |  |
| Haplic luvisols ferric | % |  | 250 m |  |
| Haplic luvisols | % |  | 250 m |  |
| Haplic planosols dystric | % |  | 250 m |  |
| Haplic planosols eutric | % |  | 250 m |  |
| Haplic solonchaks sodic | % |  | 250 m |  |
| Haplic solonchaks | % |  | 250 m |  |
| Haplic vertisols eutric | % |  | 250 m |  |
| Haplic vertisols | % |  | 250 m |  |
| Soil organic carbon density ((depth 5 cm) | kg/m3 |  | 250m |  |
| Palmer drought severity Index | index | TerraClimate:  https://climate.northwestknowledge.net/TERRACLIMATE/index_directDownloads.php | 25Km | The Palmer Drought Severity Index (PDSI) is a measure of drought conditions that is widely used in hydrology and climatology. It is based on the balance between precipitation and evaporation and is calculated using a combination of meteorological data and hydrological models. The PDSI is used to assess drought conditions over time and to identify areas that are at risk of drought. |
| Soil moisture | m^3/m^3 |  | 25 km | The data are derived from a combination of observations and model simulations, and are available at daily, monthly, and annual time scales. |
| Run offs | Mm/yr |  | 25 Km |  |
| Climate Hazards Infrared Precipitation (Climate Hazard Group) | Mm | Climate Hazard Center:  http://chg.geog.ucsb.edu/data/chirps | 5 km | The data are derived from a combination of satellite observations and ground-based weather station measurements and are available from 1981 to the present. |
| Enhanced vegetation index | index | **AfSIS:**  http://africasoils.net/services/data/remote-sensing/land | **30 m** | The data are derived from satellite observations and are available at monthly intervals. The EVI is a normalized difference index that uses visible and near-infrared wavelengths to estimate the amount of photosynthetically active vegetation present in an area. It is often used to monitor and assess the health of vegetation over time and can be used for a variety of applications, including agriculture, land use planning, and natural resource management. |
| Livestock - Gridded Livestock density | average number of animals per km2 | HAVARD, Dataverse:  https://dataverse.harvard.edu/dataset.xhtml?persistentId=doi:10.7910/DVN/GIVQ75 | 10 km | Animal numbers are distributed homogeneously with equal densities within their census polygons (areal weighting) to provide spatial data layers free of any assumptions linking them to other spatial variables [4]. |

**References**

1. Trabucco A, Zomer RJ. Global High-Resolution Soil-Water Balance. - figshare. Dataset. 2019 [cited 5 Dec 2022]. Available: https://doi.org/10.6084/m9.figshare.7707605.v3

2. New M, Lister D, Hulme M, Makin I. A high-resolution data set of surface climate over global land areas. Clim Res. 2002;21: 1–25. doi:10.3354/cr021001

3. Franks S, Storrey JC, Rengarajan R. The new Landsat Collection-2 Digital Elevation Model. 2020 [cited 10 Dec 2022]. doi:10.3390/rs12233909

4. Gilbert M, Nicolas G, Cinardi G, Van Boeckel TP, Vanwambeke SO, Wint GRW, et al. Global distribution data for cattle, buffaloes, horses, sheep, goats, pigs, chickens and ducks in 2010. Sci Data. 2018. doi:10.1038/sdata.2018.227
